# FAIR data needed to liberate hepatitis B virus (HBV) from the catch-22 of neglect

**DOI:** 10.7189/jogh.09-010310

**Published:** 2019-06

**Authors:** Philippa C Matthews

**Affiliations:** 1 Nuffield Department of Medicine, Medawar Building for Pathogen Research, University of Oxford, Oxford, UK; 2 Department of Microbiology and Infectious Diseases, Oxford University Hospitals NHS Foundation Trust, John Radcliffe Hospital, Oxford, UK; 3 Oxford Biomedical Research Centre, John Radcliffe Hospital, Oxford, UK

Hepatitis B virus (HBV) is currently estimated to be responsible for 290 million chronic infections globally, accounting for >800 000 deaths each year [1]. Infection is endemic among some of the world’s poorest and most vulnerable communities, exemplified by many settings across the African subcontinent. International Sustainable Development Goals set out the challenge of combating viral hepatitis, with specific targets developed by the World Health Organisation (WHO) for the elimination of these infections as a public health threat by the year 2030 [2]. For a crisis of such magnitude, with focused global targets now in place, we start from a position of surprising ignorance. A sustained lack of economic, scientific, clinical and political investment offer some explanation as to why HBV can aptly be considered a neglected tropical disease (NTD) [3]. The morbidity and mortality arising from HBV infection are predictable consequences of poverty, and we should be mindful of neglected peoples as much as of a neglected pathogen [4].

There are still gaps in our understanding of the biology of this unusual partially double-stranded DNA retrovirus [5]. For Africa, even basic observational epidemiology is poorly described, transmission routes are not properly characterised, the immunological correlates of vaccine-mediated immunity are uncertain, and there are substantial gaps in describing the distribution and clinical impact of drug resistance mutations [3,6,7]. We are further hindered by stigma and discrimination, which can be potent barriers to individual diagnosis and treatment, impede progress at population level, inhibit political advocacy, and interfere with collection of reliable data [3,8]. All of these attributes of HBV infection vary across a continent as diverse and heterogenous as Africa; we cannot assume that a focused insight derived from one setting is universally applicable.

**Figure:**
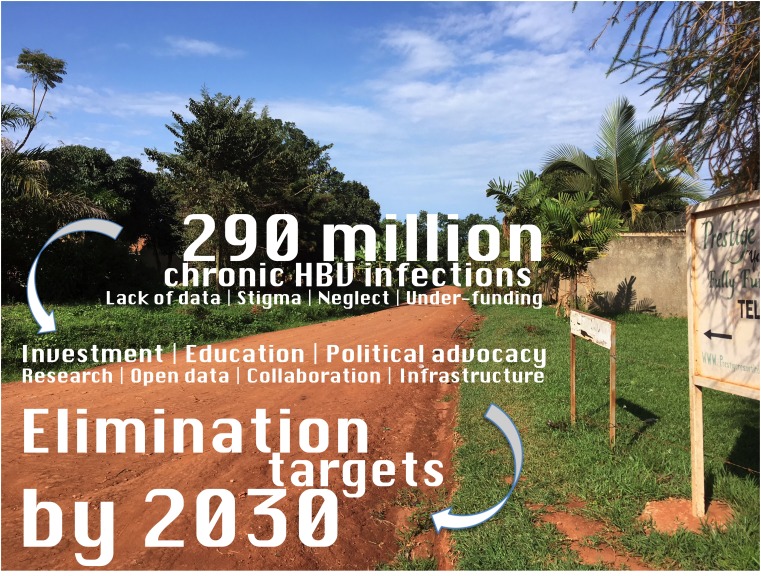
Photo: The road to HBV elimination (photo from the collection of Philippa Matthews, used with permission)

At present, attempts to enhance and develop progress for HBV can almost universally be criticised for their poor foundations. It is too easy for this inhibitory influence to become the status quo, and herein lies the catch-22: neglect begets neglect. This phenomenon is exemplified by an analysis of NTD research from Nigeria, reporting that most data are ‘published in journals with no known impact factor’ [9]. While impact factor should be applied with caution as a measure of quality, the metric in this case certainly reflects a neglected body of work, lacking quality assurance, that does not receive recognition or generate re-use.

The WHO ‘roadmap’ that directs us towards elimination goals for NTDs recognises the need for generating and sharing high quality data [10]. Developing a similar roadmap for HBV should include a strong mandate for producing and publishing new insights, empowering the development and sharing of evidence-based observations, resources, and recommendations. Areas where there are gaps in current knowledge need to be identified and systematically advanced, with publication through routes that incorporate scientific integrity and robust peer review. This approach is enshrined in the “FAIR” data principles, reflecting the need for data to be **F**indable, **A**ccessible, **I**nteroperable and **R**eproducible [11].

We must also be willing to recognise and accept deficiencies in preexisting data. This means that - while caveats are made explicit and transparent, and potential limitations are recognised – we are nevertheless able to foster an environment in which advances can emerge despite a backdrop of chronic neglect. Areas of uncertainty must be allowed to benefit from an open cycle of data sharing, critique and refinement. We are otherwise at risk of continuing to suppress progress in a field that so urgently needs to flourish if we are to make meaningful strides towards 2030 targets.
